# Research on the disaster bearing characteristics system and capacity depth disaster response curves in urban underground three dimensional spaces

**DOI:** 10.1038/s41598-025-19649-5

**Published:** 2025-10-13

**Authors:** Yan Wang, Peng Liu, Shaozhi Chu, Jin Lin, Hongwei Liu, Tao Ma

**Affiliations:** 1 Nanjing Hydraulic Research Institute, Nanjing, 210098 China; 2 State Key Laboratory of Hydrology, Water Resources and Hydraulic Engineering Science, Nanjing, 210098 China

**Keywords:** Underground space, Floods, Index system, Escape time, Capacity-depth-disaster curve, Disasters, Environmental sciences, Hydrology

## Abstract

The rapid development of urban underground space utilization, coupled with the increasing frequency of extreme precipitation events, has heightened the vulnerability of underground spaces to flood disasters, rendering flood prevention a critical challenge in underground infrastructure planning. While existing research predominantly focuses on pre-disaster early warning systems, in-disaster evacuation protocols, and post-disaster recovery strategies for underground flooding, systematic investigations into the disaster-bearing characteristics of underground spaces and the interrelationships among storage capacity, water depth, and disaster mechanisms remain notably deficient. To address these gaps, this study consolidates methods for identifying flood disaster-bearing characteristics in urban three-dimensional spatial contexts and establishes a comprehensive indicator system encompassing external flooding, underground waterlogging, and disaster-specific attributes. By defining the safe evacuation water depth at ground-level exits as a critical control threshold, we derive site-specific safe evacuation times and propose a methodology for constructing capacity-depth-disaster (C-D-D) curves to quantify flood resilience. These contributions provide a scientific foundation for abstracting representative underground space models, analyzing volume-dependent flood response mechanisms, and guiding integrated surface-underground disaster prevention planning at regional scales, thereby bridging theoretical research and practical decision-making in urban flood risk management.

## Introduction

Against the backdrop of rapid urbanization, the three dimensional urban underground space has emerged as a critical solution for relieving surface land scarcity and enhancing urban functional intensification. In recent years, Urban Underground Space(UUS) development has expanded globally, with increasing density of subterranean infrastructure such as metro systems, underground commercial complexes, parking garages, and utility tunnels—now constituting essential components of modern urban frameworks. However, climate change has led to a growing frequency of extreme weather events. In particular, the Intergovernmental Panel on Climate Change (IPCC)^1,2^ has repeatedly emphasized the rising intensity and frequency of extreme precipitation, which significantly heightens the risk of urban waterlogging and pluvial flood disasters^3^.

In recent years, numerous major flood events in underground spaces have underscored a global, frequent, and high-risk trend^4–7^. For example, in July 2021, Zhengzhou, China, was hit by a historically unprecedented rainstorm, during which floodwaters rapidly inundated the Metro Line 5 tunnel, resulting in 14 fatalities—a stark warning about the vulnerability of underground urban spaces. Similarly, in September 2021, Hurricane Ida triggered torrential rainfall in New York City, USA, flooding more than 50 subway stations, disrupting multiple lines, and paralyzing the public transportation system—highlighting that even developed cities face flood resilience deficiencies in their underground systems. In July 2023, a vehicular tunnel in Cheongju, South Korea, experienced severe backflow and flooding due to prolonged heavy rainfall, tragically claiming 13 lives and exposing gaps between urban drainage infrastructure and emergency response mechanisms. Due to inherent structural constraints, underground spaces are generally characterized^8^ by low elevation, limited drainage capacity, high enclosure, and restricted access, making them particularly vulnerable to backflow and inundation during extreme rainfall events. Compared to surface flooding, underground flooding is often more abrupt and destructive, with diverse intrusion pathways^9^—including entrances, ventilation shafts, and tunnel joints. Constraints on individual evacuation time and space^10^, together with the complexity of rescue operations^11^, pose significant challenges for accurately estimating disaster losses. Once a disaster occurs, these factors can lead to severe consequences.

Against this backdrop, an increasing number of scholars have shifted their research focus toward the resilience of UUS in the context of flood disasters. Resilience, originally derived from the fields of ecology and engineering, refers to the capacity of a system to cope with unexpected disturbances^12^. It has since been widely applied in multidisciplinary contexts such as urban governance and disaster risk management. The City Resilience Framework, jointly developed by the Rockefeller Foundation and Arup, defines urban resilience as “the capacity of individuals, communities, or systems to survive, adapt, and grow in the face of chronic stresses and acute shocks.”^13^At its core, resilience emphasizes a system’s ability to withstand disturbances, recover rapidly, and evolve adaptively. Renfei He et al.^14^, in the context of flood resilience in UUS, divides resilience into four successive stages: Pre-flood preparedness, Reaction, Recoverability, and Post-flood adaptability. These stages collectively describe the capacity of underground systems to resist, respond to, and adapt to flood hazards throughout the entire disaster cycle.

International research has focused on pre-disaster early warning and forecasting, in-disaster evacuation and refuge, and post-disaster emergency recovery^15]– [16^. Huang et al.^17^investigated the resilience of urban underground infrastructure—particularly metro systems—under multi-hazard scenarios, systematically assessing their vulnerability and recovery capacity from structural and network perspectives under compound disasters such as floods and earthquakes. Bi et al.^18^proposed an analytical framework for evaluating the resilience of urban rail transit systems under flood conditions. By applying a “stress testing” approach, they simulated the impact of key node failures on the overall system functionality, identifying high-vulnerability nodes and critical paths. Hong Ling et al.^19^developed an emergency evacuation simulation model for metro stations, improving upon the traditional potential field and social force models. Marta Borowska-Stefańska et al.^20^proposed optimized strategies and routes for orderly evacuation under flood scenarios, emphasizing improvements in evacuation efficiency and safety. Jiazhi Zhong et al.^21^integrated rainfall intensity, terrain conditions, and underground space distribution characteristics to establish a methodological framework for assessing the inundation risk of urban underground spaces under extreme rainfall events. Huang Yong et al.^6^ analyzed the causes of the fatal Zhengzhou Metro Line 5 flooding incident (“7.20 Event”) from both spatial and institutional/behavioral dimensions and proposed targeted strategies for optimizing emergency response mechanisms. From the perspective of urban resilience, Liu et al.^12^assessed the potential role of underground space in disaster prevention and mitigation. They constructed a multi-indicator comprehensive evaluation system, suggesting that rational planning and utilization of underground spaces can significantly enhance a city’s capacity to resist and rapidly recover from natural disasters. Zhichao Chen et al.^22^developed a coupled aboveground–underground urban logistics network model to evaluate the reliability and vulnerability of urban underground space systems under extreme rainfall–flooding and cascading failure scenarios.

Existing research on flood disasters in urban underground spaces predominantly focuses on single-dimensional risk assessments or localized disaster prevention technologies. However, there is a lack of a systematic indicator system integrating the three-dimensional morphological characteristics, external flood intrusion mechanisms, and internal resilience capacity of these spaces. Significant gaps remain, particularly in the quantitative analysis of dynamic response relationships among capacity-water depth-disaster impacts and the multi-tiered characteristics of disaster-bearing entities.

Based on the refined measurements of complex underground space structures using advanced semi-enclosed underground structural surveying instruments (i.e., backpack-mounted 3D laser scanners), and considering the geographical information attributes, flood characteristics, and disaster features of various types of underground spaces, a comprehensive flood indicator system was developed. This study establishes the relationship between surface water depth and underground space inflow by analyzing the correlation between surface water accumulation depth and the intrusion rate of floodwaters into underground areas. Taking the critical escape water depth at surface-level exits as a control condition, the study incorporates Japan’s underground water intrusion escape theory^23^ to estimate the safe evacuation time for specific underground spaces. In addition, the study explores the variation process of rising water depth within underground spaces, and based on localized extreme precipitation scenarios, calculates the capacity–depth–disaster response curves under 100-year, 50-year, and 20-year return periods. Furthermore, a composite urban waterlogging risk classification method is developed using accumulated water depth and flood velocity, enabling risk level assessment of urban underground spaces.

## Study area

Tongzhou District is located in the southeastern part of Beijing, bordering Chaoyang District and Daxing District to the west, connected to Hebei Province across the Chaobai River to the east, and adjacent to Tianjin Municipality to the south. It serves as Beijing’s sub-center. Tongzhou District lies in the downstream regions of the North Canal (Beiyunhe) and Chaobai River basins, with an average annual precipitation of 546.8 mm. Due to the low-lying terrain and the convergence of multiple rivers within the region, it is prone to external flooding and internal waterlogging disasters^24^. By collecting and processing relevant data, essential datasets on topographic elevation, land surface conditions, rainfall data, and municipal drainage networks in Tongzhou District were acquired.

### Topographic elevation

**Fig. 1:**
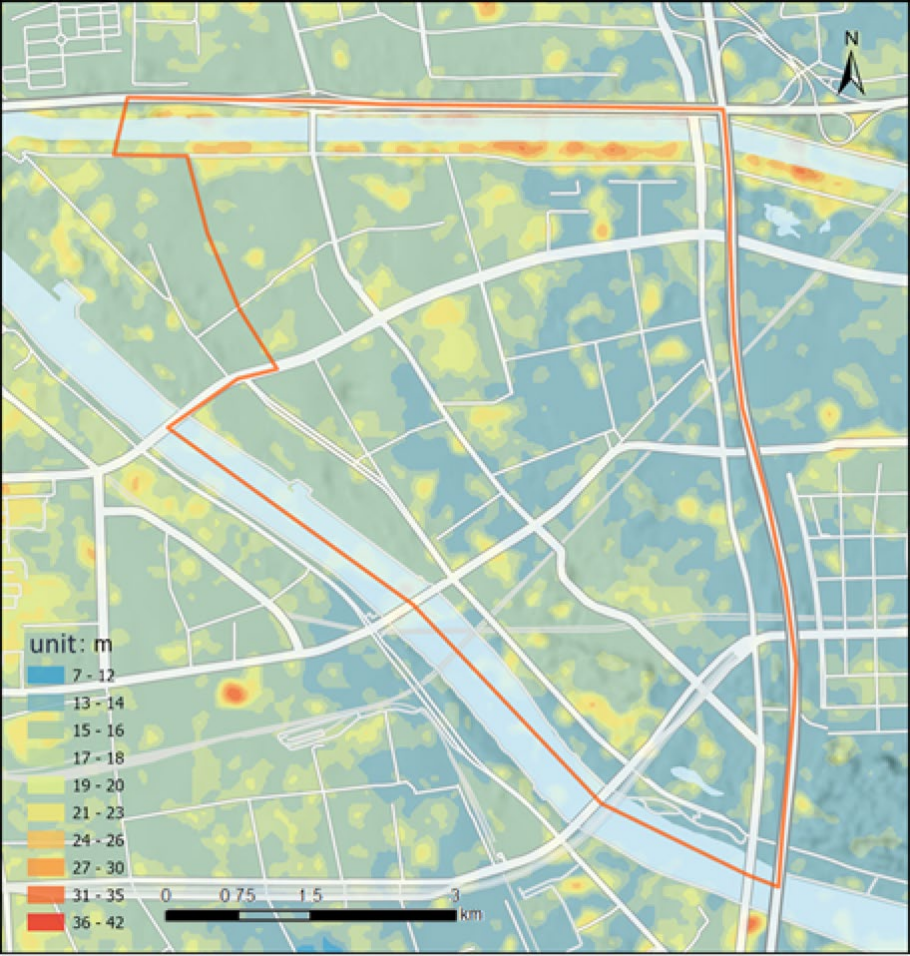
Topographic Elevation Map of Subzone 01, Beijing Urban Sub-center(Created Using ArcGIS Desktop 10 Simplified Chinese Edition; URL: https://www.esri.com/en-us/home).

Topographic elevation data is obtained from the national fundamental geographic information datasets of the target city, including Digital Elevation Models (DEMs), Digital Line Graphics (DLGs), and Ground Control Points (GCPs). In cases of data scarcity, drone-based remote sensing survey data or other corrected remote sensing DEMs may also serve as the basis for analytical calculations. Figure 1 illustrates the topographic elevation map of Subzone 01 in the Beijing Urban Sub-center, collected for this study.

### Underlying surface conditions

**Fig. 2:**
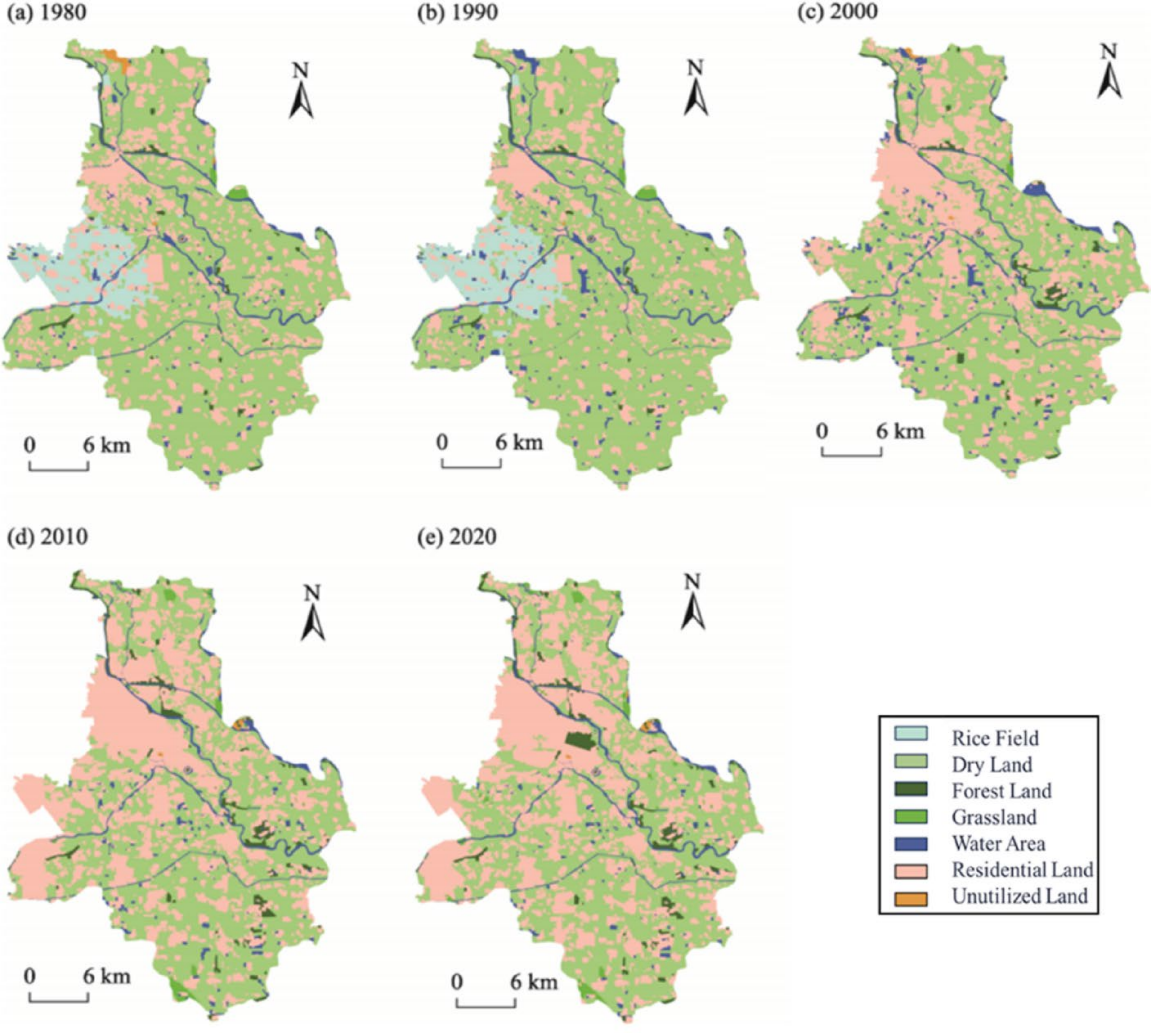
Land Use Type Distribution Maps of Tongzhou District, Beijing, Across Historical Periods(Created Using ArcGIS Desktop 10 Simplified Chinese Edition).

Analysis of land use type distribution maps (Fig. 2) collected for Tongzhou District from 1980 to 2020 reveals two distinct urbanization phases:

**Phase 1 (1980–2010): accelerated urbanization:**

Significant land use changes occurred, with urban residential areas expanding markedly, cultivated land (primarily paddy fields and drylands) decreasing, and previously unused land in the northwestern region being developed. A linear urban expansion pattern emerged along the Sixth Ring Road and the Grand Canal. By 2010, the land use composition shifted from predominantly agricultural in 1980 to a near-equal balance between cultivated and residential areas.

**Phase 2 (2010–2020): stabilized urbanization:**

Urban boundaries became relatively fixed, with land use types stabilizing. Forestland concentration and scale increased around the northern Grand Canal, forming a northwest-to-southeast gradient of urban-rural differentiation and intensive land development.

The Beijing Urban Sub-center, Tongzhou’s most representative urbanization zone, exhibited parallel spatiotemporal evolution:

#### 1980–2010

Rapid expansion of residential areas along the Grand Canal and Sixth Ring Road, accompanied by modest forestland growth and reduced water bodies.

#### 2010–2020

Urban expansion decelerated, land use stabilized, forestland aggregated into large contiguous areas, while grassland and unused land remained sparsely distributed.

### Rainfall data

**Fig. 3:**
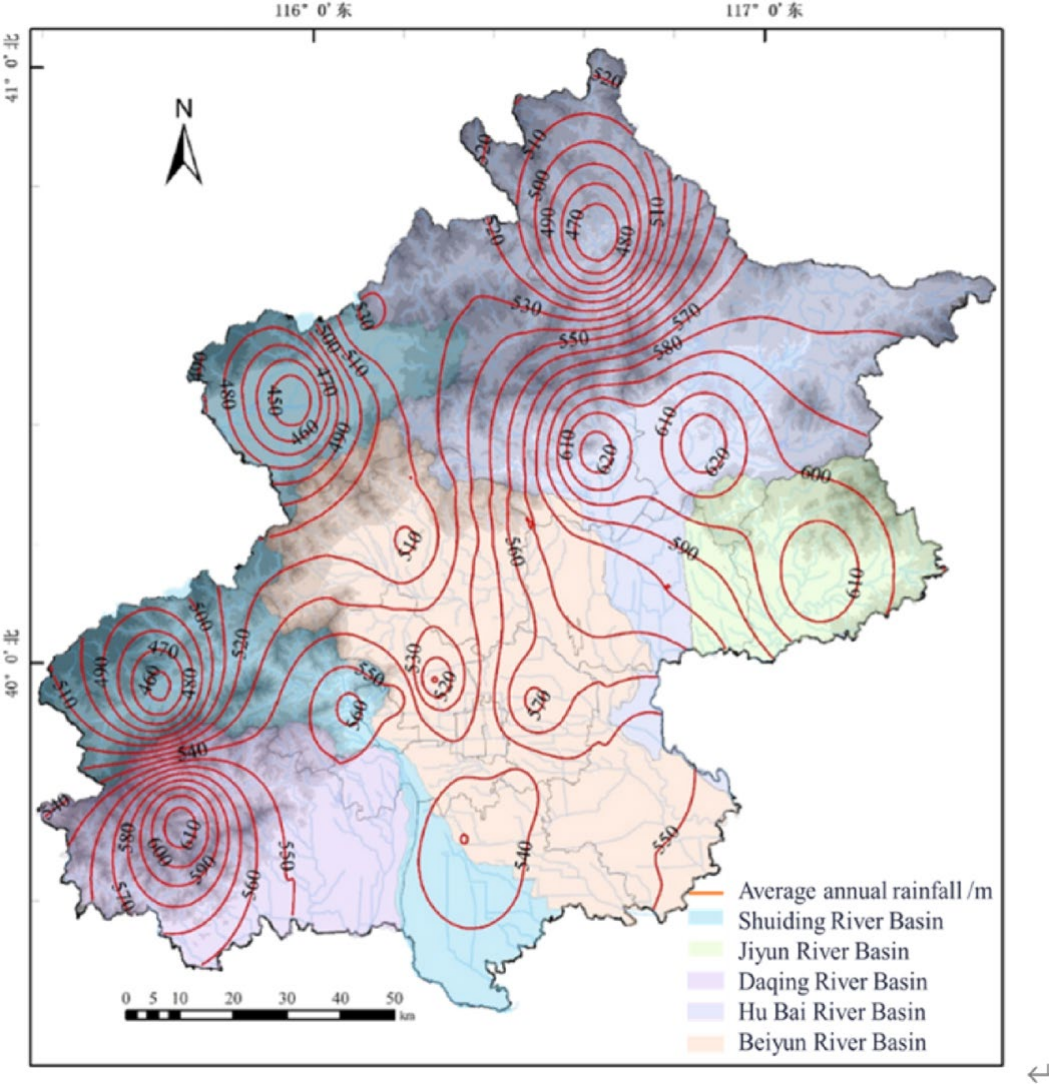
Meteorological Station Network and Multi-Year Average Rainfall Spatial Distribution Map of Beijing (Created Using ArcGIS Desktop 10 Simplified Chinese Edition).

The historical climate observation data can be obtained from the national reference climate stations. Daily rainfall sequence data from 1980 to 2019 were obtained from 20 representative meteorological stations across Beijing(Figure 3).

### Municipal drainage network

**Fig. 4:**
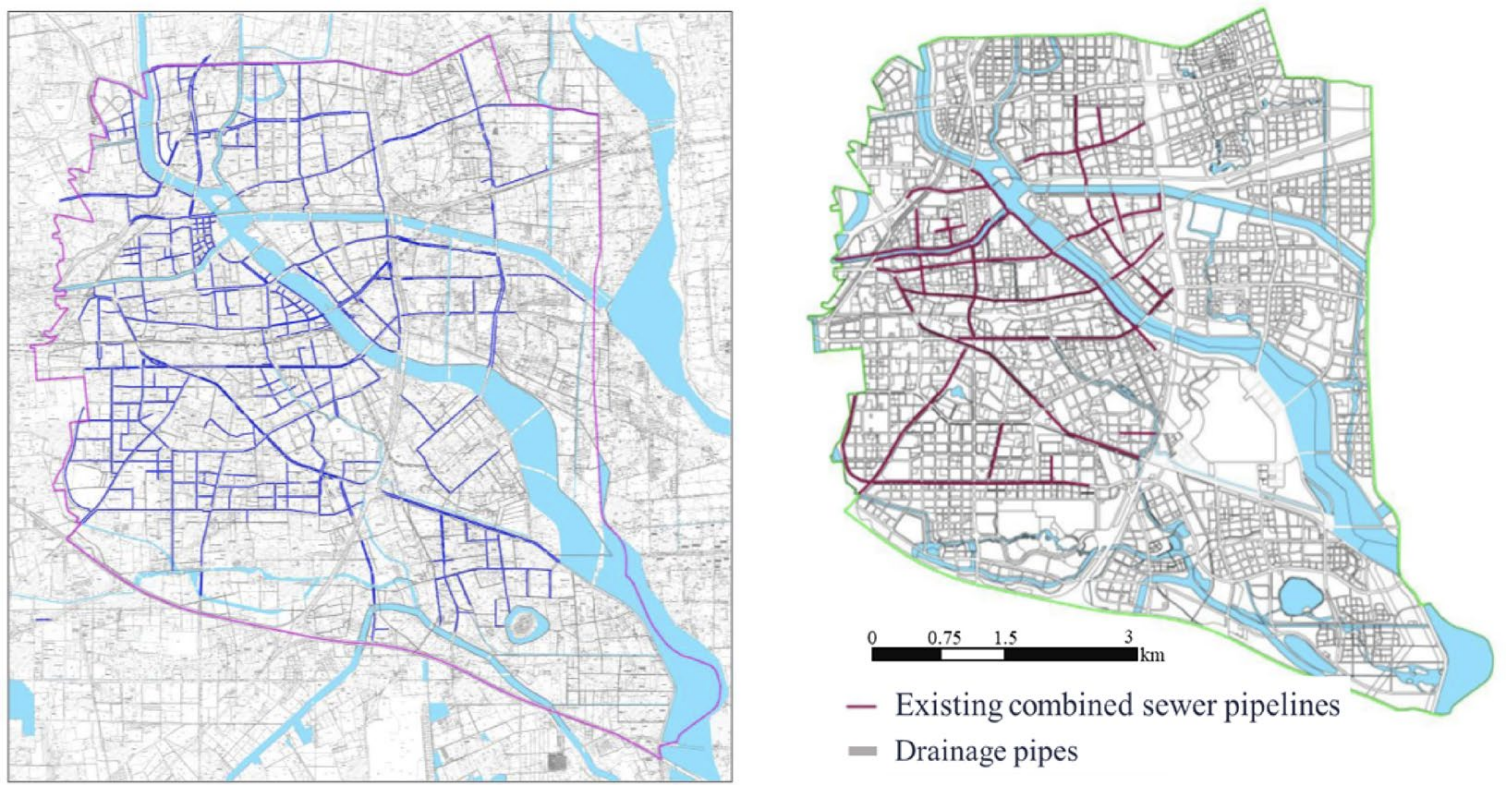
Schematic diagram of existing stormwater pipelines and combined sewer pipelines in the beijing urban sub-center (Created Using ArcGIS Desktop 10 Simplified Chinese Edition).

According to the survey results of existing stormwater pipelines in the Beijing Urban Sub-center, the area currently encompasses 23 natural rivers, including 7 flood control channels and 16 drainage waterways. Municipal roads within the Sub-center span approximately 565 km, with stormwater pipelines progressively constructed alongside road development over the years. As of the end of 2016, data provided by the Tongzhou District Water Authority indicates that the total length of operational stormwater pipelines in the Sub-center reached approximately 226 km. Due to connections with upstream or adjacent wastewater pipelines, a portion of the stormwater pipelines now function as combined sewer systems. Currently, the total length of combined sewer pipes in the Sub-center measures approximately 66.8 km(Fig. 4).

The record-breaking “23·7” torrential rainstorm event in Beijing in 2023 primarily concentrated its rainfall over Tongzhou District, with the highest precipitation recorded at Wangjiayuan Reservoir Station in Changping District. This was the greatest rainfall recorded in the Beijing area in 140 years of instrumental observation^25^.

Through the analysis of the current situation and planning environment of Beijing’s sub-center, the urban three-dimensional spaces were categorized into three main types based on their spatial characteristics, flood risk features, and disaster attributes for further analysis. These categories are defined according to their openness: open, enclosed, and semi-enclosed. The three-dimensional flood-bearing bodies of a typical megacity are summarized as follows: road-tunnel-bridge areas (open flood-bearing bodies), underground garages, civil air defense works, and underground structures (enclosed flood-bearing bodies), and underground shopping malls and metro stations (semi-enclosed flood-bearing bodies).

**Fig. 5:**
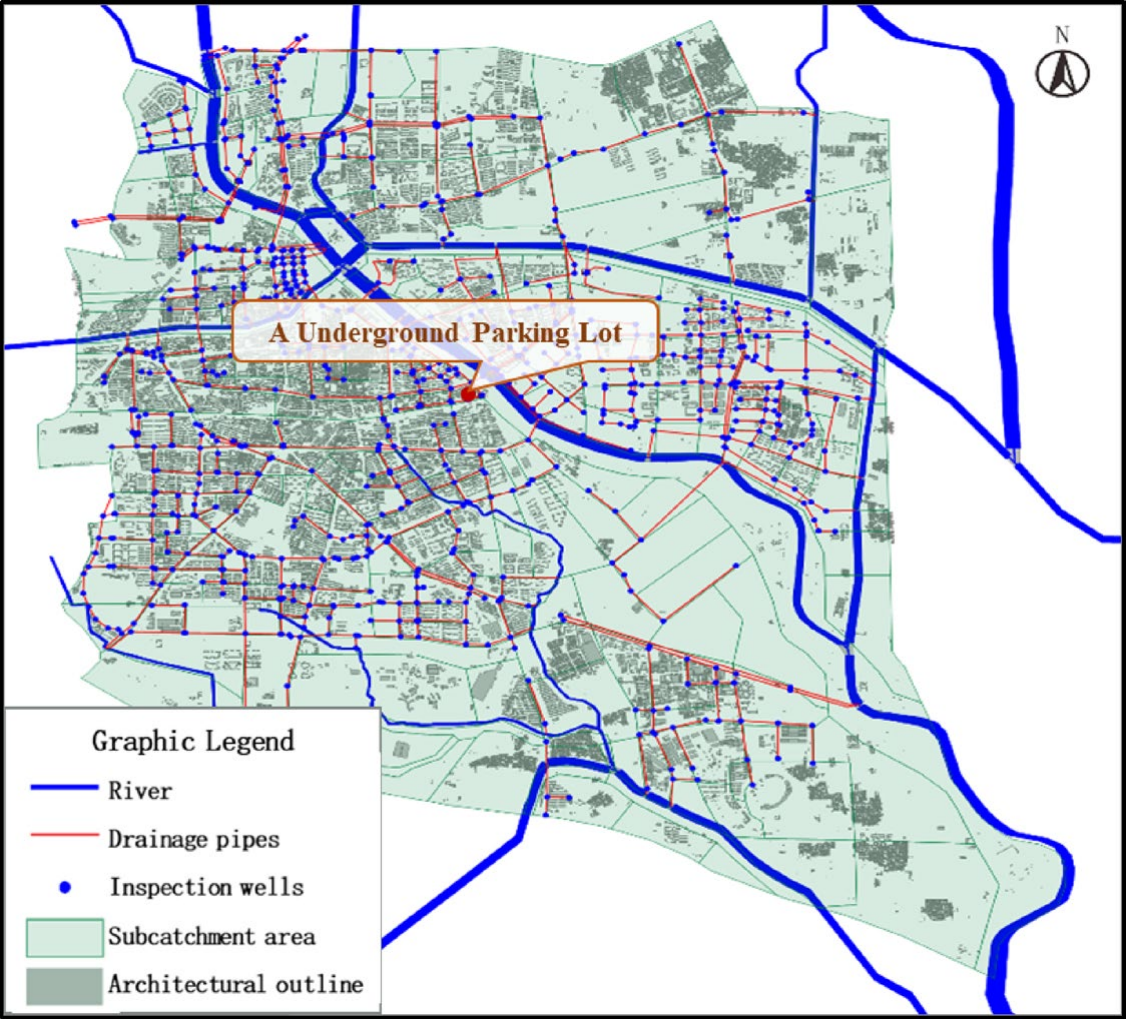
Typical Underground Space Location Map of Beijing’s Urban Sub-Center. (Created Using ArcGIS Desktop 10 Simplified Chinese Edition)

According to the flood risk map of Beijing released by the Beijing Water Authority, a few high-risk areas (with waterlogging depths exceeding 60 cm) are scattered within the study area. Based on topography and field data collection conditions, two typical underground spaces were selected for field investigation: the enclosed flood-bearing body of underground parking lot A (single-story)(Figure 5). Using backpack-mounted 3D laser scanners, three-dimensional laser point cloud data of these typical underground spaces were acquired.

## Research methodology

### Underground space flood indicator system

Based on the fundamental characterization parameters of urban flood-prone three-dimensional spaces, this study establishes a characteristic indicator system for urban flood disaster-bearing entities in three-dimensional spaces. This system addresses external surface flooding, underground waterlogging, and disaster attributes through comparative analysis of global research status on urban flood disasters, integration of disaster prevention experiences from urban three-dimensional spaces, examination of actual historical cases of urban spatial flood disasters, review of existing research progress, and reference to existing standards and regulations. The process involved systematic identification of disaster-causing indicators related to urban three-dimensional space flooding.

The selection principles for indicators were primarily considered from the following aspects:

- Comprehensiveness Principle: Indicators should comprehensively reflect the impact severity of underground space flooding through multidimensional considerations, ensuring authentic representation of disaster effects.
- Representativeness Principle: Selected indicators must demonstrate direct correlation with underground space flood occurrences, effectively characterizing disaster features with minimal yet typical parameters that exemplify essential attributes.
- Scientific Principle: Indicators should be grounded in scientific rationality, objectively revealing correlations in underground space flooding while providing theoretical support and scientific basis for flood simulation analysis.
- Accuracy Principle: Indicators must precisely demonstrate their relationship with underground space flooding, avoiding redundant parameters that fail to accurately reflect disaster mechanisms while eliminating subjective influences in selection.

Considering the principles of comprehensiveness, representativeness, scientificity and accuracy, the underground space flood indicator system for external flood, underground waterlogging and disaster characteristics was constructed in this paper (Table 1).

**Table 1:** Underground space flood indicator system.

Primary Indicator	Secondary indicator	Tertiary indicator
External flood	Terrain altitude	
	Underlying surface conditions	Land use types
		Hydraulic structures
		Other Structures
	Rainfall	
	Municipal pipe network	
Underground waterlogging	Attributes of underground space entrance and exit	Entrance elevation
		Number of entrance and exit
		Entrance width
		Slope of entrance and exit
	Morphological attributes of underground space	Area of underground space
		Height of underground space
		Layers of underground space
		Underground space connectivity
	Drainage capacity of underground space	Scale of gathering wells
		Number of gathering wells
		Installed capacity of drainage pump station
		Number of drainage pump stations
Disaster characteristics	Disaster-bearing body attributes	Population density
		Vehicle density
		Economic value
		Lifeline Engineering Information
	Disaster indicator index	Ground water depth, flow rate
		Escape time of underground space
		Disaster-causing rainfall intensity in underground space
		Rising process of underground water
		Capacity-depth-disaster response curve
	Disaster rescue capability	Flood prevention and control plan

### The variation process of water depth rise in underground spaces

The rising rate of water level in underground spaces is directly proportional to the entrance width and inversely proportional to the available storage area. This relationship reflects a fundamental hydraulic mechanism in flood dynamics: a wider entrance allows more floodwater to enter per unit time, and a smaller storage area results in a faster rise in water level.

**Fig. 6:**
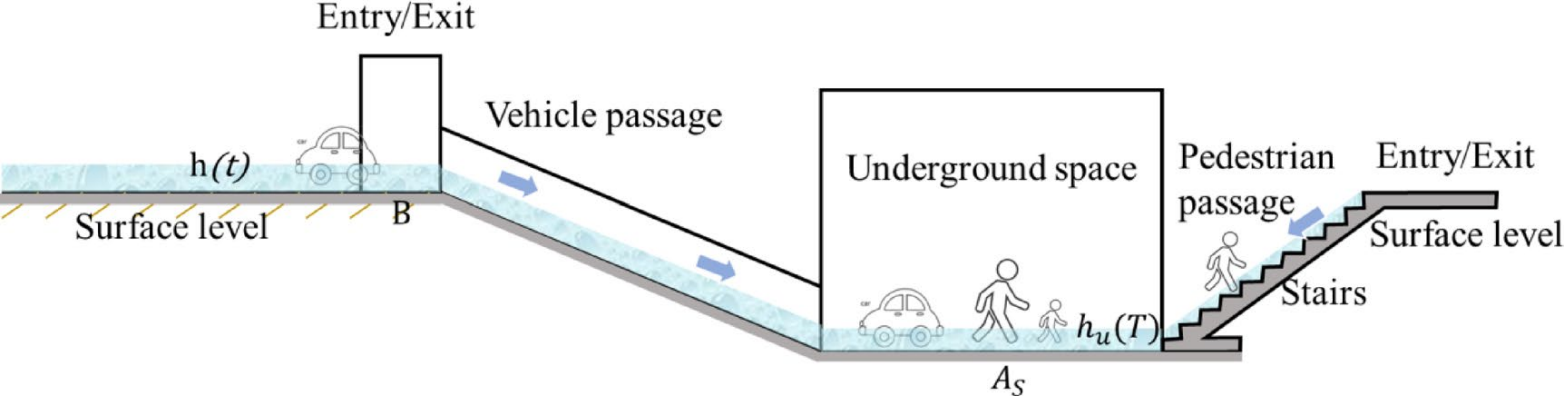
Conceptual diagram of surface water inflow and inundation process.

As shown in the Fig. 6, during intense rainfall events, surface water rapidly accumulates in low-lying areas, with the surface water depth increasing over time as $\:h\left(t\right)$. Once the surface water exceeds the threshold elevation of the underground entrance, gravity-driven flow occurs through staircases or ramps, channeling water into the underground space. Given that underground spaces are typically enclosed or semi-enclosed, once inflow begins, the internal water level $\:{h}_{u}\left(T\right)$ rises rapidly.

The ground surface and the interior of underground spaces (such as underground parking lots) are typically open or flat areas, where the water depth is relatively large and the flow velocity is relatively low. Therefore, the flow in these regions is considered subcritical. In contrast, stairwells or ramp entrances usually function as narrow channels, where water rapidly descends due to elevation differences. In such areas, the water depth is shallow, the velocity is high, and the flow is inertia-dominated, corresponding to a supercritical flow condition.

in underground space.

According to the experimental results on stairwell physical models by Taisuke Ishigaki et al.^28^, the relationship between the inflow through staircases into underground spaces and the surface water depth at the entrance is given by the following equation:

1 $${q_u}=1.98{h^{1.621}}$$

Where:$\:{q}_{u}$—represents the unit-width flood discharge at the entrance of the underground space(m^3^/s/m);$\:h$—The surface water depth at the entrance(m).

The total volume of floodwater intruding into the underground space at T seconds is

2 $$V\left( T \right)=\mathop \smallint \limits_{0}^{T} 1.98Bh{\left( t \right)^{1.621}}dt=1.98B\frac{{v_{t}^{{1.621}}}}{{2.621}}{T^{2.621}}$$

Where: $\:V\left(T\right)$ —The total floodwater volume entering the underground space at cumulative time (m³);$\:B$ — Width of the entrance staircase to the underground space (m);$\:h\left(t\right)$—Surface water depth (m); $\:{v}_{t}$—surface water depth rise rate(m/s). T (capital letter) denotes the cumulative time during the rising process, while t (lowercase letter) refers to an instantaneous time step in the calculation.

By dividing the inflow volume by the available storage area $\:{A}_{s}$ of the underground space, the water depth of underground flooding at $\:T$ seconds is:

3 $${h_u}\left( T \right)=V\left( T \right)/{A_s}=1.98B\frac{{v_{t}^{{1.621}}}}{{2.621{A_s}}}{T^{2.621}}{\text{~}}$$

The rising velocity of underground water levels is:

4 $${u_t}={h_u}^{{{\prime }}}\left( T \right)=1.98v_{t}^{{1.621}}{T^{1.621}}B/{A_s}$$

Where: $\:{u}_{t}$ —The rising velocity of underground water levels (m/s);$\:{A}_{s}$—area of the underground space(m^2^).

The relationship between the rising velocity of underground water depth and the rising velocity of surface water depth is:

5 $${u_t}/v_{t}^{{1.621}}=1.98{T^{1.621}}B/{A_s}$$

The purpose of including Eq. (5) is to explicitly show the functional dependence between the rising velocity of underground water depth $\:{u}_{t}\:$and the rising velocity of surface water depth $\:{v}_{t}$.

**Fig. 7:**
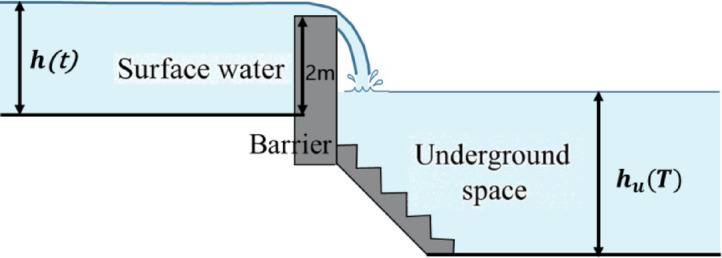
Schematic diagram of floodwater intrusion into the underground space with a 2 m barrier at the entrance.

The flood flow rate invading the underground space per unit time varies with the surface water depth, and the rise rate of the water level in the underground space also differs. For a fixed surface water depth, the water level in the underground space rises at a constant rate(Fig. 7). Furthermore, the rise rate of the water level in the underground space is related to the width of the underground space entrance and the floor area. The rise rate of the water level in the underground space is proportional to the entrance width and inversely proportional to the floor area of the underground space.

### Calculation method for underground space evacuation time

Flood intrusion into underground spaces poses a severe threat to human life if evacuation is not timely. On one hand, rapidly rising water levels can lead to drowning. On the other hand, if surface water at the entrance reaches a critical depth, excessive water flow velocity and depth on staircases may prevent individuals from safely evacuating underground spaces to the surface.

The time required for safe evacuation from underground spaces during flood intrusion consists of five components. This evacuation time framework is derived from prior studies^23^. The five components that constitute the time required for safe evacuation from underground spaces during flood intrusion are as follows:

- **Flooding risk awareness time**: The time taken for individuals to recognize the danger of flooding.

6 $${t_1}=\hbox{min} \,\,({\text{The time when the water depth on the ground surface reaches 1}}0{\text{ cmin}})$$

According to the Trial Standard for Flood Safety Evacuation Assessment in Underground Space^23^(p. 4), a water depth of 10 cm is used to determine the “evacuation initiation threshold.” Based on field experiments conducted by Japan’s disaster prevention research institutions, when the water depth within an underground space reaches 10 cm, most individuals can clearly perceive the presence of floodwater and begin to react. This depth is considered the minimum threshold at which individuals are able to sense inundation and initiate preliminary evacuation behavior.

- **Evacuation awareness communication time**: The time required to effectively communicate the need for evacuation to individuals in the affected area.

7 $$\:t_{2} = \frac{{\sqrt {A_{S} } }}{{30}} + 3\,\left( {\min } \right)$$

Where: $\:{A}_{S}$——area of the underground space, m^2^.

- **Walking time on flat ground within the underground space**: The time it takes for individuals to travel across flat surfaces within the underground space to reach the evacuation route.

8 $$\:t_{3} = \frac{l}{{\alpha \:v}}\,\left( {\min } \right)$$

**Fig. 8:**
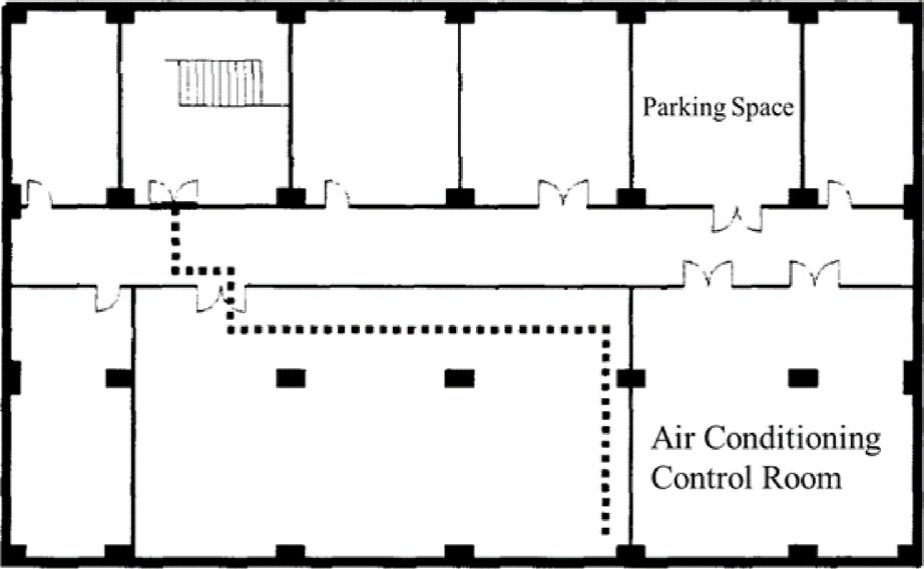
Schematic Diagram of the Walking Distance $\:\varvec{l}$.

Where: $\:l$——walking distance (In, Fig. 8), m; $\:v$——standard walking speed on flat ground (60 m/min). It is stipulated that the walking speed is zero when the water depth is 70 cm. Assuming that the water depth during walking is$\:\:{h}_{u}$cm, at this time the impact factor is $\:\alpha\:=1-{h}_{u}/70$.

Martínez-Gomariz et al.^26^conducted experimental studies to analyze the factors influencing pedestrian stability under urban pluvial flooding conditions, with a particular focus on the critical conditions for loss of balance under various combinations of water depth and flow velocity. Most experiments showed that adults with a height of approximately 160–170 cm found it nearly impossible to maintain stable walking when the water depth reached 70 cm in the absence of flowing water. Therefore, 70 cm is regarded as the theoretical upper limit of safe walking depth, beyond which the walking speed is assumed to be zero.

- **Time to pass through the underground entrance of the evacuation staircase**: The time required for individuals to navigate through the staircase entrance located underground.

9 $$\:t_{4} = \frac{{\sum \: PA_{S} }}{{\sum \: NB^{,} }}\,\left( {\min } \right)$$

Where: P——population density of the underground space, in people per square meter, which is determined according to the function of the underground space;

N——effective flow coefficient (= 90) ;This parameter represents the number of people that can pass through one meter of exit width per unit time (persons/min·m), and is used to calculate the time required for occupants to evacuate through an exit. In the Provisional Evacuation Safety Verification Method for Underground Flooding^27^, a standard value of *N* = 90 persons/(min·m) is adopted. This value is derived from extensive human evacuation experiments and empirical studies based on fire evacuation scenarios, and serves as a conservative benchmark for evacuation time assessment. B’——width of the underground entrance of the evacuation staircase(m).

- **Time to reach the surface exit via the evacuation staircase**: The time taken for individuals to ascend the staircase from the underground entrance to the surface-level exit.

10 $$\:t_{5} = \frac{{\lambda \:}}{{\beta \:v}}\,\left( {\min } \right)$$

Where: $\:\lambda\:$——distance of the staircase(m);

$\:v$——walking speed on the staircase(30 m/min).According to the literature^27^, walking is considered physically impossible when the overtopping water depth on staircase treads reaches 30 cm, as the corresponding hydrodynamic force index exceeds 1.5. Specifically, the hydrodynamic force index represents the ratio between the horizontal hydrodynamic force exerted by floodwater on a pedestrian and the pedestrian’s body weight. Specifically, the hydrodynamic force index represents the ratio between the horizontal hydrodynamic force exerted by floodwater on a pedestrian and the pedestrian’s immersed body weight. At this threshold, pedestrians are likely to lose balance due to significant water force acting on the legs, making safe evacuation infeasible. In addition, the International Building Safety Association (IBSA) also defines that “**walking becomes hazardous when step ponding exceeds 0.3 m**,” reinforcing this safety threshold.

It is stipulated that it is impossible to walk when the water depth on the surface of the staircase steps reaches 30 cm. Assuming that the water depth on the surface of the staircase steps during escape is $\:y$ cm, at this time the impact factor β = 1 - y / 30.

Therefore, the time required for people to evacuate when the underground space is invaded by flood is.

11 $$\:T_{u} = t_{1} + \frac{{\sqrt {A_{S} } }}{{30}} + 3 + \frac{l}{{60\left( {1 - \frac{{h_{u} }}{{70}}} \right)}} + \frac{{\sum \: PA_{S} }}{{\sum \: NB^{,} }} + \frac{{\lambda \:}}{{30\left( {1 - \frac{y}{{30}}} \right)}}\,\,\left( {\min } \right)$$

When staircases are used as the control condition, it is required that individuals in the underground space can safely evacuate via the staircases. In this case, the water depth at the surface-level staircase exit must remain below the critical escape water depth of 0.3 m, satisfying the condition $\:{T}_{u}\le\:{T}_{s}$, where $\:{T}_{s}$is the time it takes for surface water to reach the critical escape depth of 0.3 m. Considering extreme conditions, it can be assumed that:

12 $$\:t_{1} + \frac{{\sqrt {A_{S} } }}{{30}} + 3 + \frac{l}{{60\left( {1 - \frac{{h_{u} }}{{70}}} \right)}} + \frac{{\sum \: PA_{S} }}{{\sum \: NB^{,} }} + \frac{{\lambda \:}}{{30\left( {1 - \frac{y}{{30}}} \right)}} \le \:T_{s} \,\,\left( {\min } \right)$$

Based on the experimental relationships and graphical results provided in the *Provisional Evacuation Safety Verification Method for Underground Flooding*^27^, the flow depth on staircase treads under various surface water depths was calculated using empirical formulas that relate inflow discharge and elevation difference. The results indicate that when the surface water depth is 30 cm, the water depth on the stair treads is approximately 10–15 cm.

Therefore, it can be assumed that the water depth on the stair treads remains constant at a depth . Among the terms, only the fourth term, $\:{h}_{u}$, implicitly includes the unknown $\:{T}_{s}$, where the basement water depth is related to the surface water rise rate, and the surface water rise rate is related to the safe evacuation time $\:{T}_{s}$. Assuming the surface water rises at a constant rate, denoted as $\:{v}_{t}$ (cm/min), according to the experimental results of the stair physical model by Japanese scholar Ishigaki Yasushi^28^, the critical surface water depth for safe evacuation from the stairway is 30 cm. Therefore, we have:

13 $$\:t_{1} \:T_{s} = \frac{{30}}{{v_{t} }}\,\,\left( {\min } \right)$$

When the awareness time is controlled by the surface water depth, consider the above equation:

14 $$\:t_{1} = \frac{{10}}{{v_{t} }} = \frac{{T_{s} }}{3}\,\,\left( {\min } \right)$$

As described in "The variation process of water depth rise in underground spaces", when the surface water reaches the critical evacuation depth, the corresponding water depth in the underground space can be derived from Eq. (3):

$$\:{h}_{u}=1.98B\frac{{\left(\frac{{v}_{t}}{6000}\right)}^{1.621}}{2.621{A}_{S}}{\left(60{T}_{s}\right)}^{2.621}\left(m\right)=\frac{198B}{2.621{A}_{S}}{\left(\frac{1}{200{T}_{s}}\right)}^{1.621}{\left(60{T}_{s}\right)}^{2.621}\left(cm\right)$$

15 $$\:=\frac{643.8B}{{A}_{S}}{T}_{s}\left(cm\right)$$

Where:$\:{T}_{s}---$The time required for evacuation during flood intrusion into underground spaces(min); B — Width of the entrance staircase to the underground space, in meters (m)༛h(t)—Surface water depth. $\:{v}_{t}$—Surface water depth rise rate(m/s);

By substituting Eqs. (13) and (14) into Eq. (15), the formula for safe evacuation time is obtained:

16 $$\:T_{s} - \frac{l}{{40\left( {1 - \frac{{9.2B}}{{A_{S} }}T_{s} } \right)}} = \frac{{\sqrt {A_{S} } }}{{20}} + 4.5 + \frac{3}{2}\frac{{\sum \: PA_{S} }}{{\sum \: NB^{,} }} + \frac{{\lambda \:}}{{20\left( {1 - \frac{y}{{30}}} \right)}}\,\,\,\left( {\min } \right)$$

According to the above equation, the safe evacuation time Ts for the underground space can be obtained:

17 $$\:T_{s} = \frac{1}{2}\left( {\frac{{40}}{a} + b} \right) \pm \:\frac{1}{2}\sqrt {\left( {\frac{{40}}{a} + b} \right)^{2} - \frac{{160b + 4l}}{a}} \,\left( {\min } \right)$$

$$\:\text{W}\text{h}\text{e}\text{r}\text{e}：a=40\times\:9.2B/{A}_{S},\:b=\frac{\sqrt{{A}_{S}}}{20}+4.5+\frac{3}{2}\frac{\sum\:P{A}_{S}}{\sum\:N{B}^{，}}+\frac{\lambda\:}{20\left(1-\frac{y}{30}\right)}$$

In addition, taking a single-layer underground space as an example, the evacuation time empirical formula was combined with the local sensitivity analysis method to quantify the impact of parameters such as underground space area, entrance width, exit width, the walking distance of the most disadvantaged individual, population density, and assumed submerged water depth on staircases on evacuation time.

- **Parameter selection**:

Based on the evacuation time empirical formula, six parameters, classified into two types, were selected. The first type relates to the attributes of the underground space, including underground space area, entrance width, and exit width. The second type involves assumed parameters, such as the walking distance of the most disadvantaged individual, population density, and assumed submerged water depth on staircases.

- **Determination of variation range**:

In the local sensitivity analysis, the impact of parameters is determined by changing one input factor at a time while keeping all other factors constant. The greater the deviation between parameter perturbation and its initial value, the lower the reliability of the analysis results. Referring to previous studies^29^, parameter variations were set at 50%, 70%, 90%, 110%, 130%, and 150% of the default values^30,31^ to evaluate the impact of parameter changes on the results.

- **Determination of Target Variable**:

The purpose of this study is to quantify the impact of parameters such as underground space area, entrance width, exit width, the walking distance of the most disadvantaged individual, population density, and assumed submerged water depth on staircases on evacuation time. Therefore, evacuation time was selected as the target variable.

### Calculate the flood capacity-depth response curve based on the local heavy rainfall pattern

#### Design rainfall Estimation under different return periods

The annual average rainfall and extreme precipitation indices in Beijing exhibit significant spatial variability, particularly between mountainous and plain areas. The study area is located in the eastern part of the Beijing plain. For the design rainfall calculation, 14 meteorological stations situated in plain areas with elevations below 100 m were selected from 20 typical meteorological stations as reference points for the analysis.

- Design rainfall parameters and calculation.

The average maximum 24-hour areal rainfall over the study area was estimated using the Thiessen polygon method. For different rainfall durations, parameters including the coefficient of variation (Cv), skewness coefficient (Cs), the ratio Cs/Cv, frequency factor (Kp), and the rainfall reduction coefficient (n) were determined with reference to the *Beijing Hydrological Manual* (hereinafter referred to as the “Hydrological Manual”) (see Table 2).Based on the above rainfall data and parameters, the design rainfall intensities for return periods of 100 years, 50 years, and 20 years were calculated for durations of 1 h, 3 h, 6 h, 12 h, and 24 h (see Table 3**)**.

**Table 2:** Table of design rainstorm parameters in Tongzhou district, Beijing.

Parameter	1 h			6 h			24 h		
C_s_/C_v_	3.5			3.5			3.5		
C_vt_	0.5			0.6			0.69		
H/mm	42.5			74			106.72		
Return period	20-year	50-year	100-year	20-year	50-year	100-year	20-year	50-year	100-year
K_p_	1.99	2.42	2.74	2.2	2.76	3.2	2.39	3.09	3.63
n	—	—	—	0.94	0.93	0.91	0.94	0.92	0.91

**Table 3:** Design rainfall calculation results for Tongzhou district, Beijing.

Duration	Rainfall depth for return period (mm)		
	20-Year	50-Year	100-Year
1 h	84.58	102.85	116.45
3 h	156.60	194.11	223.00
6 h	162.80	204.24	236.80
12 h	244.71	311.66	363.73
24 h	255.06	329.77	387.40

- Design rainfall temporal distribution:

Based on the 100-year return period design rainfall depths for durations of 1 h, 6 h, and 24 h in the Beijing plain area, the temporal distribution was determined with reference to the recommended synthetic rainfall patterns in the *Beijing Hydrological Manual*. The maximum 1-hour rainfall was assigned to the 23rd time step, and the total 24-hour design rainfall depth was distributed across all time steps according to the specified distribution ratios. The proportion of rainfall assigned to each time step was then calculated. The results are shown in the Table 4.

**Table 4:** Design rainfall and net rainfall depths for different return Periods.

Time interval(min)	Time interval (mm)			Net rainfall (mm)		
	*P* = 1%	*P* = 2%	*P* = 5%	*P* = 1%	*P* = 2%	*P* = 5%
1	0.00	0.00	0.00	0.00	0.00	0.00
2	0.00	0.00	0.00	0.00	0.00	0.00
3	0.00	0.00	0.00	0.00	0.00	0.00
4	0.00	0.00	0.00	0.00	0.00	0.00
5	24.51	20.83	16.10	15.33	10.55	6.47
6	24.51	20.83	16.10	15.33	10.55	6.47
7	12.68	10.77	8.33	7.93	5.45	3.34
8	12.68	10.77	8.33	7.93	5.45	3.34
9	10.14	8.62	6.66	6.34	4.36	2.68
10	0.00	0.00	0.00	0.00	0.00	0.00
11	0.00	0.00	0.00	0.00	0.00	0.00
12	0.00	0.00	0.00	0.00	0.00	0.00
13	0.00	0.00	0.00	0.00	0.00	0.00
14	0.00	0.00	0.00	0.00	0.00	0.00
15	13.22	11.23	8.68	8.26	5.69	3.49
16	8.59	7.30	5.64	5.37	3.70	2.27
17	37.00	31.45	24.31	23.14	15.92	9.76
18	7.27	6.18	4.78	4.54	3.13	1.92
19	34.11	28.99	22.41	21.33	14.68	9.00
20	14.78	12.56	9.71	9.24	6.36	3.90
21	7.96	6.76	5.23	4.98	3.42	2.10
22	24.13	20.51	15.85	15.09	10.38	6.37
23	116.45	98.97	76.51	72.81	50.10	30.72
24	39.37	33.46	25.87	24.62	16.94	10.39

#### Flood hydrographs for different return periods

The design flood hydrographs were computed using the drainage modulus method and the Nash instantaneous unit hydrograph method for flood frequencies of *P* = 1%, *P* = 2%, and *P* = 5%. In the drainage modulus method, the catchment area F and slope J were extracted and calculated from a 30-meter resolution DEM. Parameters such as lithological conditions, design runoff depth R, and design groundwater table depth were obtained from the *Beijing Hydrological Manual*. The calculation results required are presented in the following Table 5.

**Table 5:** Peak drainage flow under different Frequencies.

Frequency (%)	Drainage modulus [m³/(km²·s)]	Drainage Discharge(m³/s)
1	1.88	321.81
2	1.33	227.31
5	0.84	144.24

In the calculation using the Nash instantaneous unit hydrograph method, the parameters $$\:n\left(number\:of\:linear\:reservoirs\right)$$$$\:k\left(storage\:coefficient\right)$$ and the S-curve values were all obtained from relevant charts in the Beijing Hydrological Manual (Table 6**)** .Net rainfall was estimated using the equal-division infiltration method, in which infiltration losses are subtracted uniformly from each time step such that the sum of net rainfall depths over all time intervals equals the total runoff depth. The computed net rainfall depth for each time step is summarized in the Table 4.

**Table 6:** Design flood calculation results using the instantaneous unit hydrograph Method.

*n*、k parameters	Frequency(%)	Peak flood discharge (m³/s)	Time lag between rainfall and flood peak (h)
*n* = 3.5 k = 1.5	1	309.66	11
	2	213.08	11
	5	130.66	11

Based on the comparison between the results obtained from the drainage modulus method and the Nash instantaneous unit hydrograph method, the deviation between the two methods remains within 10% for rainfall frequencies of 1% and 2%, with a slightly higher deviation of 10.39% observed under the 5% frequency scenario **(**Table 7). Therefore, the overall calculation results are considered reliable.

**Table 7:** Comparison of calculation results between the drainage modulus method and the instantaneous unit hydrograph method.

Frequency(%)	Peak discharge by drainage modulus method(m³/s)	Peak discharge by Nash IUH method(m³/s)	Deviation (%)
1	321.81	309.66	3.92%
2	227.31	213.08	6.68%
5	144.24	130.66	10.39%

#### Calculate the flood-carrying capacity–water depth response of the underground space based on local storm rainfall characteristics

Based on the runoff volume estimated using the Nash instantaneous unit hydrograph method for the study area, and in combination with the contributing area of the underground catchment, the total runoff can be further converted into runoff depth per unit area. This represents the accumulation of surface runoff over a specific period on a unit area basis, providing a quantitative measure of the runoff intensity within the catchment.

Based on the experimental results of the stair physical model by Ishigaki Yasushi et al.^28^, the relationship between the flood flow rate invading the underground space through the stairway and the water depth at its entrance is given by the equation $\:{q}_{u}=1.98{h}^{1.621}$, where $\:{q}_{u}$is the unit width flood flow rate at the underground space entrance (m³/s/m), and $\:h\:$is the surface water depth at the underground space entrance (m). This study takes into account the actual conditions of the residential area when calculating the A underground garage, assuming the presence of a 0.2-meter-high barrier or sandbags on the ground to block water under extreme rainfall scenarios.

After calculating the flood flow rate at the underground space entrance, the water depth process curve for the underground space is obtained by combining the floor area of the underground space. The water inflow process and the corresponding capacity-depth-disaster curves for the underground space under the 100-year, 50-year, and 20-year return periods are derived, along with the disaster times corresponding to different disaster thresholds.

To enhance the readability of the equations and facilitate understanding, a comprehensive Table of Symbols is provided below (Table 8). All major symbols used in the manuscript are systematically summarized with their physical meanings and corresponding units. This allows readers to quickly grasp the logical relationships in the derivations and minimizes ambiguity in the use of symbols.

**Table 8:** Table of Symbols.

No.	Symbol	Definition	Unit
1	$\:{q}_{u}$	Represents the unit-width flood discharge at the entrance of the underground space	m^3^/s/m
2	$\:h$	The surface water depth at the entrance	m
3	$\:V\left(T\right)$	The total floodwater volume entering the underground space at cumulative time	m^3^
4	$\:B$	Width of the entrance staircase to the underground space	m
5	$\:h\left(t\right)$	Surface water depth	m
6	$\:{h}_{u}\left(T\right)$	The water depth of underground flooding	m
7	$\:{A}_{s}$	Area of the underground space	m²
8	$\:{u}_{t}$	The rising velocity of underground water levels	m/s
9	$\:{v}_{t}$	Surface water depth rise rate	m/s
10	$\:{t}_{1}$	Flooding Risk Awareness Time	min
11	$\:{t}_{2}$	Evacuation Awareness Communication Time	min
12	$\:{t}_{3}$	Walking Time on Flat Ground within the Underground Space	min
13	$\:{t}_{4}$	Time to Pass Through the Underground Entrance of the Evacuation Staircase	min
14	$\:{t}_{5}$	Time to Reach the Surface Exit via the Evacuation Staircase	min
15	$\:{T}_{\text{s}}$	The time required for evacuation during flood intrusion into underground spaces	min
16	P	Population density of the underground space	persons/m^2^
17	N	Effective flow coefficient	persons/min·m
18	B’	Width of the underground entrance of the evacuation staircase	m
	$\:l$	Walking distance	m
19	$\:\lambda\:$	Distance of the staircase	–
	$\:N$	Effective flow coefficient (= 90)	–
	β	Assuming that the water depth on the surface of the staircase steps during escape is $\:y$ cm, at this time the impact factor	–
20	$\:v$	Walking speed on the staircase	m/min
21	Cv	Arameters including the coefficient of variation	–
22	Cs	Skewness coefficient	–
23	Kp	Frequency factor	–
24	n	The rainfall reduction coefficient	–

## Results analysis

- Identification of Underground Waterlogging Characteristics.

Currently, commonly used measurement techniques for surface depressions and underground structures include total station surveying and three-dimensional laser scanning. Total stations provide high-precision three-dimensional spatial data with Z-coordinate values and operate through point-by-point data acquisition. In contrast, 3D laser scanning technology captures high-density geometric coordinate data of terrain and complex object surfaces in the form of point clouds through non-contact, high-speed laser scanning. This method enables rapid and accurate acquisition of high-resolution 3D coordinate information over large surface areas of the measured objects^32^.

**Fig. 9:**
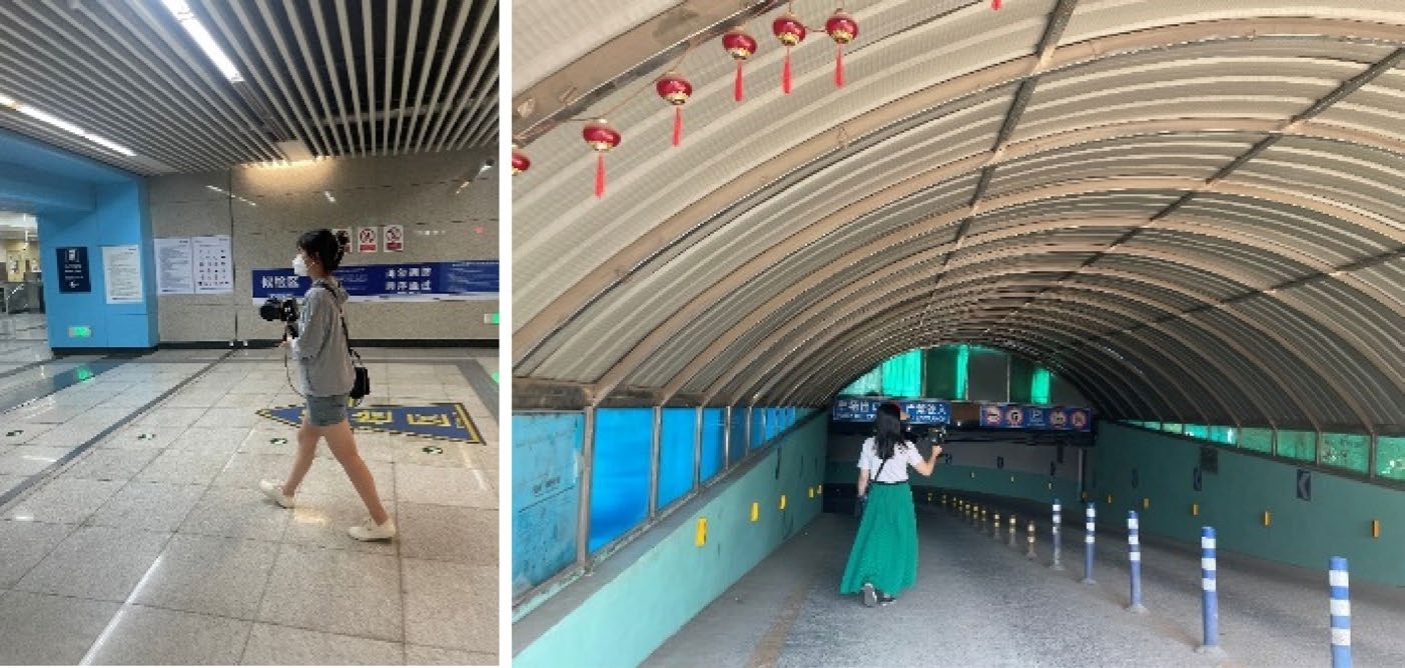
Field Investigation of Typical Underground Spaces in the Demonstration Zone (The individuals appearing in the figure are the authors involved in the field investigation.)

Field Investigation in the Beijing Urban Sub-center Demonstration Zone(Fig. 9). Field surveys were conducted using a GEO Slam handheld 3D laser scanner to measure typical underground spaces. A single operator-device unit can complete 3D point cloud field data acquisition for an underground space of approximately 4,000 m² within 10 min. No external human participants were involved in the field investigation.

**Fig. 10:**
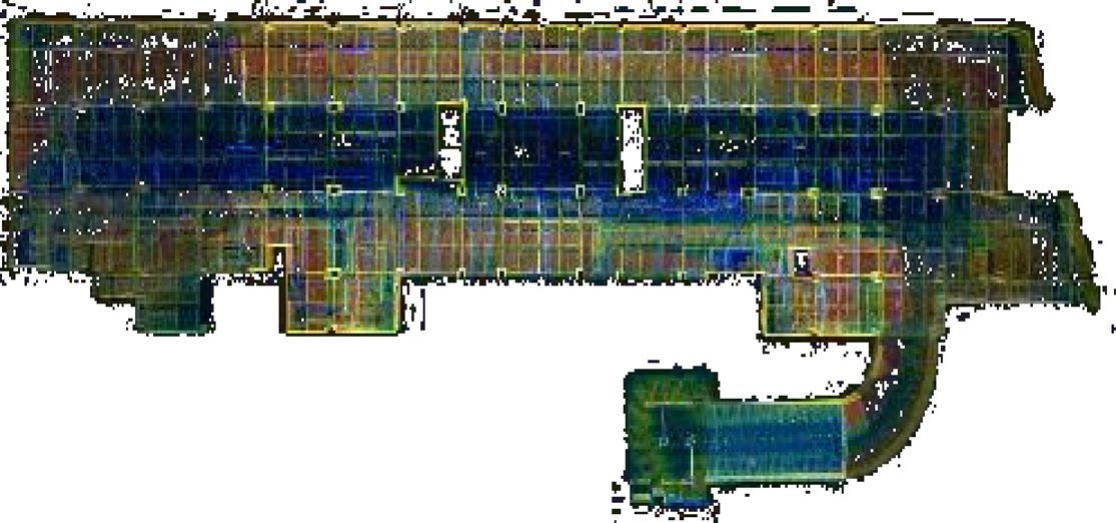
Top View of 3D laser scanning point cloud data of the underground parking lots.

The raw point cloud data acquired through LiDAR measurements requires post-processing to generate elevation model data. Due to the substantial volume of raw point cloud datasets, computational processing time typically ranges from 1 to 3 times the scanning duration (Fig. 10).

According to the results of the 3D laser scanner, import the original data into the Hub for preprocessing and calculation, and obtain the typical three-dimensional index data values of the underground space (Table 9.).

**Table 9:** Underground waterlogging Indicators-Underground parking lot A.

Primary Indicator	Secondary indicator	Tertiary indicator	Indicator value
Underground waterlogging	Entrance attributes	Entrance elevation	11.52
		Number of entrances	1
		Entrance width	7.45 m
		Entrance slope	0.40
	Morphological attributes	Underground space area	3758 m^2^
		Underground space floor height	3 m
		Number of floors	1
		Underground space connectivity	None
	Drainage capacity	Sump capacity	~ 1 m^3*^(estimated per relevant standards)
		Number of sumps	2* (estimated per relevant standards)
		Drainage pump station installed capacity	/
		Number of drainage pumps	/

* indicates estimated per relevant standards.

Attributes of disaster-bearing entities can be obtained through multiple approaches, including population heatmap data, surveillance camera footage, quantified damage assessments, stakeholder feedback, and field investigations. Emergency response planning data can be directly accessed from official sources. The following section outlines the computational methodologies employed to derive disaster-indicative indicators.

### Changes in underground space water depth rise process

The A underground parking lot is a single-level structure. Based on field investigations and 3D scanning data, the area of the underground space is approximately 127.8*29.4 m², with both the ground-level entrance and the basement exit measuring 11.6 m. The water depth rise process in Underground Space A was calculated using the method described in "The variation process of water depth rise in underground spaces".

#### Fixed surface water depth and underground space water level rise rate

According to Eqs. (14–17), when the surface water depth remains constant at 30 cm, the rise rate of the water level in the underground parking lot can be obtained from the above equation:

18 $${u_t}=\frac{{1.98{h^{1.621}}B}}{{{A_s}}}=5.58{\text{*}}{10^{ - 4}}\left( {{\text{m}}/{\text{s}}} \right)$$

#### Constantly rising surface water depth and underground space water level rise rate

**Fig. 11:**
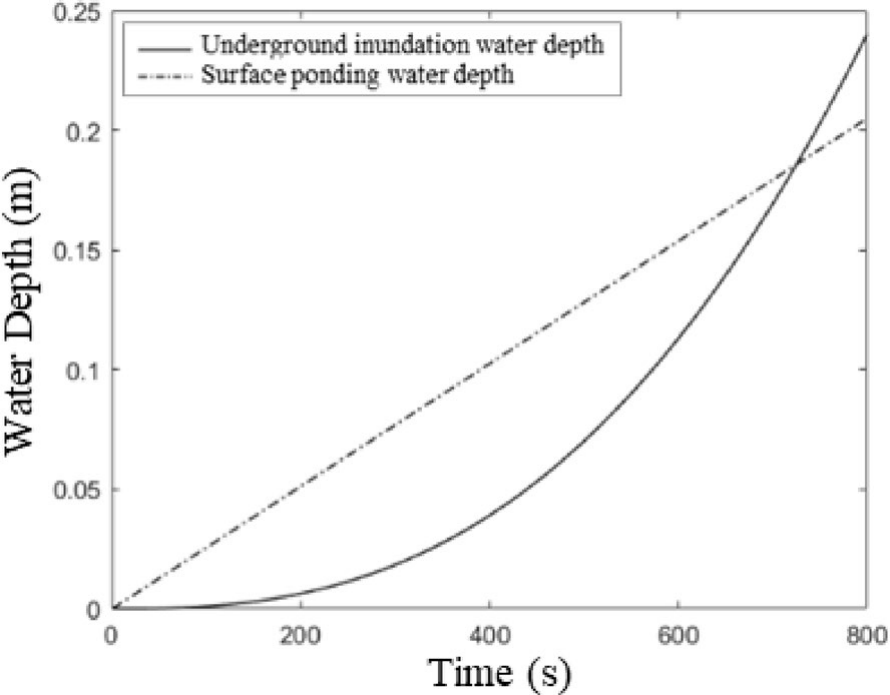
The variation process of the submerged water depth in Underground Parking Lot A and the water depth of surface ponding.

Figure 11 shows the change process of the underground flooding depth and surface water depth in the underground parking lot when the surface water rises at a constant rate of $\:{2.56\text{*}10}^{-4}(m/s)$. In particular, the underground inundation water depth is calculated using Eqs. (3)–(5), while the surface ponding water depth is given by Eq. (13).Initially, the surface water depth is greater than the underground flooding depth. When the surface water depth reaches 10 cm, the underground flooding depth in the parking lot is approximately 4 cm. As the surface water depth increases, the rise rate of the underground flooding depth in the parking lot accelerates. After about 12 min, the underground flooding depth exceeds the surface water depth.

### Estimation of evacuation time for underground space

Using the aforementioned research estimation methods, the evacuation time and the capacity-depth curves for typical areas in underground spaces A were calculated. A sensitivity analysis was also conducted on the relevant parameters.

#### A underground parking lot

The A underground parking lot consists of one floor. Based on actual surveys and scanned data, the area of the underground parking lot A_S_ is approximately 127.8*29.4 m², with a ground entrance width A of 11.6 m and an underground exit width B of 11.6 m. The stair slope length $\:{\lambda\:}_{1}$​ is approximately 30.5 m, and the most unfavorable horizontal walking distance for individuals $\:{l}_{1}\:$is approximately 111 m.

When the surface water depth is 30 cm, the water depth on the stair treads is about 10–15 cm. Assuming the water depth on the stairs $\:y$ is 10 cm, and the population density in the underground parking lot $\:{P}_{1}\:$is 0.5 persons/m^2^. The time required for evacuation at different stages is as follows:

19 $${t_1}=\frac{{{T_s}}}{3}~~({\text{min}})$$

20 $${t_2}=\frac{{\sqrt {{A_S}} }}{{30}}+3=5.04{\text{~~}}\left( {{\text{min}}} \right)$$

21 $${t_3}=\frac{l}{{\alpha v}}={l_1}/\left( {60 - 60{\text{*}}643.8{\text{*}}A{\text{*}}{T_s}/{A_S}/70} \right)\left( {{\text{min}}} \right)$$

22 $$t_{4} = \frac{{\sum PA_{S} }}{{\sum NB^{{}} }} = P_{1} *A_{S} /90/B = 2.8\,\left( {\min } \right)$$

23 $${t_5}=\frac{\lambda }{{\beta v}}=\frac{{{\lambda _1}}}{{30\left( {1 - \frac{y}{{30}}} \right)}}=2.28{\text{~}}\left( {{\text{min}}} \right)$$

By substituting the equations into formula (12), the shortest time required for people to safely evacuate from the basement is calculated as: $\:{T}_{s}$=19.5 min.

According to the empirical formula for evacuation time, a total of 6 parameters from two categories were selected for comprehensive analysis. One category is related to the attributes of the underground space, including the underground space area, entrance width, and exit width. The other category involves assumed parameters, including the most unfavorable walking distance for individuals, population density, and the assumed water depth on the stairs.

**Fig. 12:**
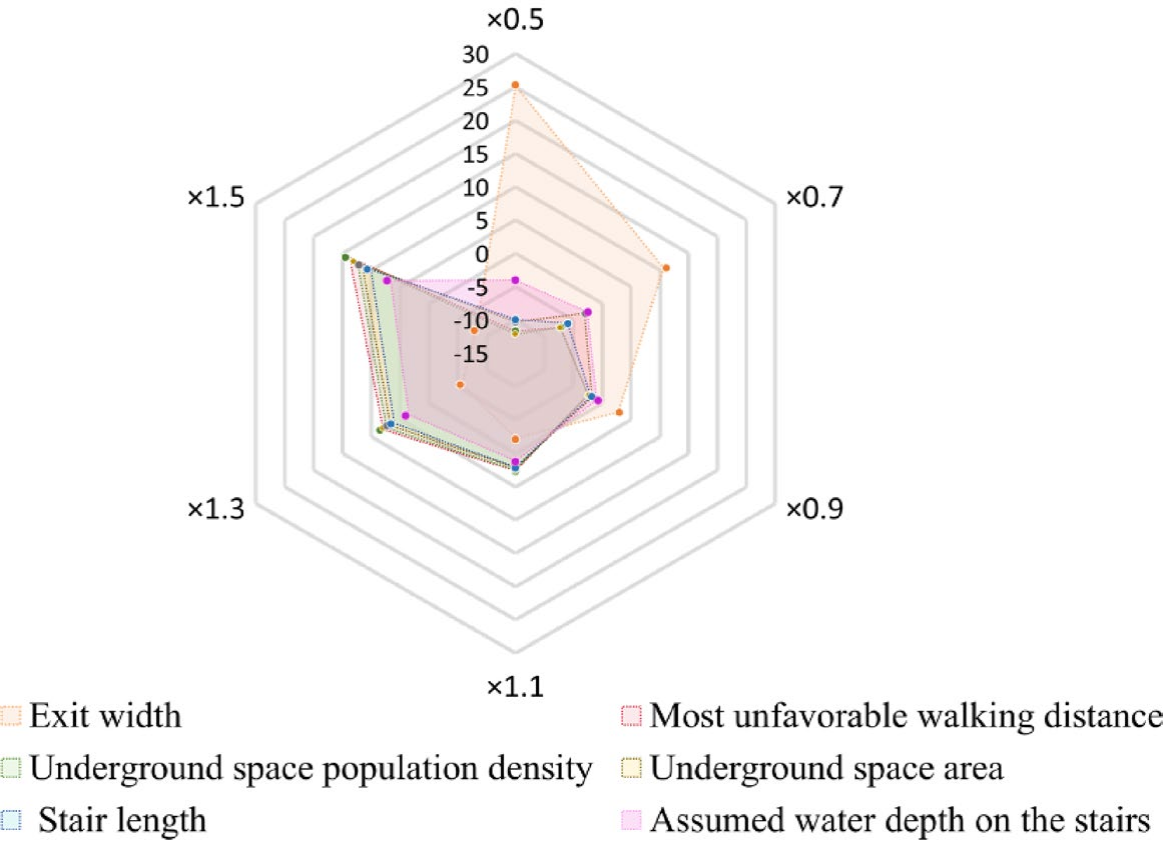
Sensitivity Analysis Results Chart of the Influence of Exit Width and Other Parameters on the Escape Time.

From the results of the sensitivity analysis (Figure 12), the importance of the parameters is ranked as follows: exit width > most unfavorable walking distance > underground space population density > underground space area > stair length > assumed water depth on the stairs. The research findings not only deepen the understanding of the empirical formula for evacuation time, but also highlight the high sensitivity of certain parameters, which are crucial for the accuracy and reliability of evacuation time calculations.

The exit width is inversely proportional to the evacuation time.

- A 50% reduction in the exit width will lead to a 25.1% increase in evacuation time.
- A 50% increase in the most unfavorable walking distance will result in a 13.8% increase in evacuation time.
- A 50% increase in population density will lead to a 12.4% increase in evacuation time.
- A 50% increase in the underground space area will result in an 11.6% increase in evacuation time.
- A 50% increase in stair slope length will lead to a 10.1% increase in evacuation time.
- A 50% increase in the assumed water depth on the stairs will result in a 6.7% increase in evacuation time.

#### Sensitivity analysis of relevant parameters

### Combining local rainfall patterns to calculate the flooding capacity-water depth response curve

At present, commonly used urban waterlogging risk assessment methods primarily rely on water depth and inundation duration as the core evaluation indicators^33^.By analyzing these two parameters, it is possible to effectively determine the extent, severity, and persistence of surface inundation, thereby providing a fundamental basis for evacuation route planning and flood-prone area delineation. However, existing flood risk assessment studies seldom incorporate surface flow velocity into the indicator system. Relevant research has demonstrated that flow velocity significantly affects pedestrian stability and the operational safety of transportation infrastructure. Therefore, flow velocity should also be considered a critical factor in flood risk evaluation and integrated into a multi-dimensional indicator system.

#### Risk determination method based on water depth

The air intake height of a typical passenger car is generally between 0.2 and 0.3 m; once water levels exceed this range, the engine is at risk of water ingress and stalling. Similarly, vehicle user manuals (e.g., from Volkswagen, Honda, etc.) explicitly warn that wading depths should not exceed 0.2–0.3 m. While SUVs or off-road vehicles typically have higher intake positions (0.4–0.6 m), wading test reports for models such as the Jeep Wrangler suggest that internal electronic systems may short-circuit when submerged. As a result, the practically safe water depth is recommended to be no more than 0.3–0.4 m. Therefore, the critical disaster-inducing water depths are set at 0.2 m for standard passenger cars and 0.3 m for SUVs and similar vehicles. For pedestrians, a water depth of approximately 0.2 m—equivalent to a child’s knee height—may prevent a child from standing up after falling. A water depth of 0.5 m is considered critical for women, while 0.7 m is the threshold for adult males beyond which stability is significantly compromised^34^.

Yifan Wang^35^, using a physical modeling approach, quantitatively assessed the risk of individual instability (being knocked down by water flow) during storm-induced coastal flooding. By considering varying water depths, flow velocities, and body postures, the study proposed critical thresholds for instability. Taking New York City as a case study, the research revealed the spatial variation in flood exposure vulnerability among urban residents and emphasized the importance of incorporating individual physical stability into flood risk management and evacuation planning.

Wang Dan et al.^36^ focusing on Xi’an City, highlighted that the current stormwater drainage design return periods are generally low. Their study pointed out that when the water depth on road surfaces exceeds 15 cm, there is a significant risk of vehicle stalling.

Li Yingxin et al.^37^, based on historical inundation data from electronic water level gauges in Zhongshan’s urban area, classified urban waterlogging risks by inundation depth: regions with h ≤ 0.05 m pose a certain flood risk; regions with 0.05 < h ≤ 0.15 m are at moderate risk; and regions with h > 0.15 m are at high risk. Xu Zongxue et al.^38^ proposed an urban waterlogging risk zoning method based on thresholds of inundation depth and duration. The risk levels are defined as follows: depth < 0.15 m as Level IV; 0.15–0.3 m with duration ≥ 30 min as Level III; 0.3–0.4 m with duration ≥ 15 min as Level II; and depth > 0.4 m as Level I.

#### Method for assessing flood flow velocity risk

Previous studies have shown that a flood flow velocity of 0.6 m/s represents the critical threshold at which a pedestrian can barely maintain balance. When the flow velocity exceeds this value, pedestrians are likely to lose stability. At velocities above 1.0 m/s, individuals may be swept away, and when flow velocities exceed 1.5 m/s, it becomes nearly impossible for a person to stand, and they are very likely to be carried off by floodwaters^39–41^.

#### Combined risk assessment method based on flow velocity and water depth

In this study, the classification of inundation risk zones adopts a pedestrian safety-based urban waterlogging risk assessment method. The method determines the Damage Factor (DF) based on water depth and combines it with flow velocity to compute the Water Disaster Risk Index (WDR)^42^. The evaluation is based on the following calculation formula:

24 $$WDR=d(v+0.5)+DF$$

In the equation, WDR represents the Waterlogging Disaster Risk Index (m^2^/s); *d* is the inundation depth (m); *v* is the flow velocity (m/s); and DF denotes the damage factor (m²/s). The value of DF primarily depends on the inundation depth. Specifically: When no inundation occurs, DF = 0 m^2^s; When inundation is present and the depth is less than 0.15 m, DF = 0.5 m²/s; When the depth ranges from 0.15 m to 1 m, DF = 1.0 m²/s; When the depth exceeds 1 m, DF = 1.5 m²/s.

In this study, the underground space is a closed structure with a single inflow entrance, and the inflow at the entrance dominates the internal hydrodynamic conditions during the initial stage. After entering the space, the water rapidly spreads and accumulates, without forming significant secondary flow paths. Moreover, the primary objective of this study is to assess flood risk in the early stages, with a particular focus on whether the inflow at the entrance poses a threat to pedestrians, vehicles, or evacuation routes. Under these conditions, using the entrance flow velocity as the parameter *v*, together with the inundation depth *d*, in the WDR calculation is considered an acceptable and practical approach for rapid risk assessment.

The floor height of the A underground parking lot is 3 m, and the maximum water inflow depth is 3 m, corresponding to an underground space volume of approximately 11,000 cubic meters. According to on-site survey data, the average elevation of the bottom of the electrical box is 1.23 m. The flood discharge of the He Pan Li Jing residential community was calculated using the instantaneous unit hydrograph method. Based on the estimated inundation area of the community, the water depth at different time steps was subsequently derived. Under extreme rainfall conditions, it is assumed that 0.2-meter-high flood barriers or sandbags are present at the ground level to block surface runoff.

According to the topography and road distribution, the watershed area is measured to be 0.1 km², with a drainage area of 53,800 m². Using the experimental results of the stair physical model by Ishigaki Yasushi et al.^28^, the relationship between the flood flow rate invading the underground space through the stairway and the water depth at its entrance is given by the equation $\:{q}_{u}=1.98{h}^{1.621}$, where $\:{q}_{u}$​ is the unit width flood flow rate at the underground space entrance (m³/s/m), and $\:h$ is the surface water depth at the underground space entrance (m).

**Fig. 13:**
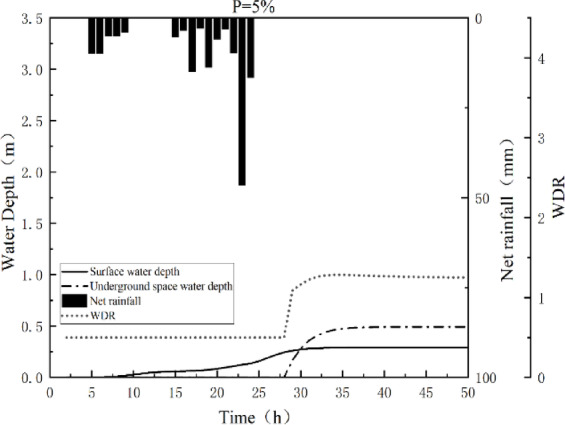
The process of water inflow into the underground space of underground parking Lot A under different frequencies.

After calculating the flood flow rate at the underground space entrance, the water depth process in the underground space is obtained by combining the floor area of the underground space. The water inflow process and the corresponding capacity-depth-disaster curves for the A underground parking lot under the 100-year, 50-year, and 20-year return periods are shown in Fig. 13.

Under the *P* = 5% rainfall frequency scenario, water began to enter the underground space at the 27th hour. By 28.3 h, the water depth reached 0.2 m, corresponding to the air intake height of a standard vehicle and the disaster threshold for children. The maximum inundation depth of 0.492 m was reached at the 41st hour. The WDR (Waterlogging Disaster Risk Index) remained at a low-risk level before hour 27, rose to the medium-risk level from hour 30 onward, and did not reach the high-risk threshold throughout the entire period.

Under the *P* = 2% scenario, inundation began at hour 24, and the 0.2-meter disaster threshold was exceeded by hour 24.5. The maximum water depth of 2.0 m was reached at hour 39. The WDR entered the medium-risk range after hour 26 and reached the high-risk level after hour 28, maintaining a high-risk status for a prolonged period.

In the *P* = 1% extreme rainfall scenario, water intrusion occurred earlier, starting at hour 22.6. By hour 23, the water depth had already surpassed the 0.2-meter threshold corresponding to standard vehicle air intakes and child hazard levels. The depth reached 1.23 m—the critical threshold for electrical equipment—at hour 25.4, and peaked at 3.052 m at hour 40. The WDR rose rapidly, entering the high-risk level after hour 28 and remaining at a high-risk state for more than ten hours thereafter, indicating significant disaster exposure and evacuation challenges for underground spaces under this scenario.

To quantitatively assess the flood-bearing capacity of underground space under varying rainfall intensities, a Capacity–Depth–Disaster (C–D–D) curve diagram was constructed based on the following three categories of information:

- Inflow process lines under different design storm frequencies:

Using design rainfall scenarios with return periods of *P* = 1%, 2%, and 5%, cumulative inflow processes into the underground space were calculated. These were represented as three inflow volume–inflow time–depth curves, shown as purple, blue, and light blue dashed lines, respectively.

- Disaster threshold markers::

Based on underground space safety standards and relevant literature, critical water depth thresholds were defined for different vulnerable targets, including: 0.2 m (ordinary cars/children, yellow marker)、0.7 m (adult male, pink diamond)、1.23 m (lowest elevation of electric equipment, black dashed line).

- Underground space storage capacity curve:

The black solid line represents the theoretical relationship between water depth and storage volume in the underground space, serving as a reference to determine whether inflow under various rainfall conditions would exceed the space’s storage capacity.

**Fig. 14:**
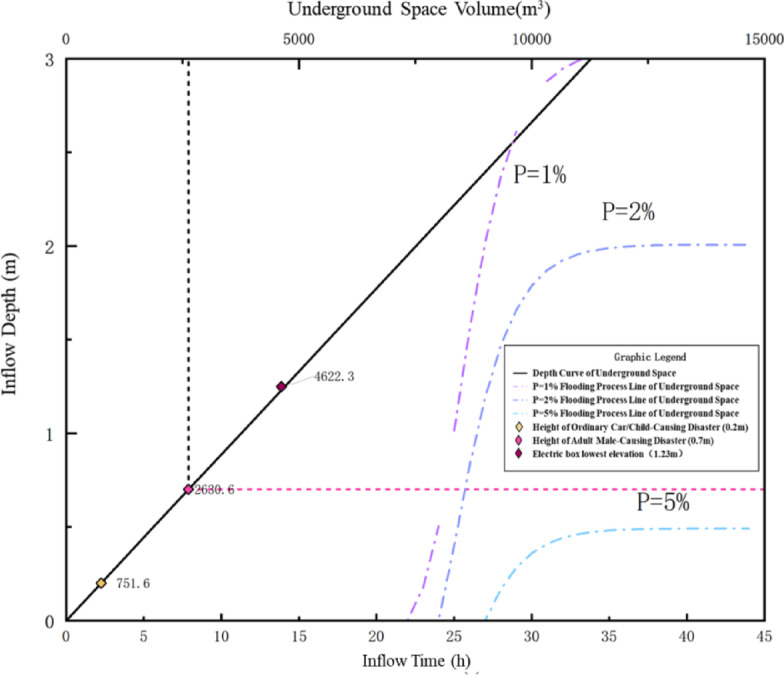
The corresponding Volume-Depth-Disaster Curves of Underground Parking Lot A under.

From the Fig. 14, it is evident that under different return period scenarios, the rate of water depth rise and the time to reach disaster thresholds vary significantly. For the adult disaster threshold (0.7 m), the corresponding inflow volume is 2630.6 m³, which is reached at 24.4 min and 25.7 min under *P* = 1% and *P* = 2% conditions, respectively, but not reached under the *P* = 5% scenario—indicating that more intense rainfall significantly increases safety risks and shortens the available evacuation window. Similarly, the disaster threshold corresponding to the lowest elevation of the electric box (1.23 m) equates to an inflow volume of 4622.3 m³, which is reached at 25.4 and 27.1 min for *P* = 1% and *P* = 2%, respectively, but again not reached for *P* = 5%, suggesting a clear flood risk to electrical equipment during extreme rainfall events. The corresponding disaster times for different disaster thresholds are shown in Table 10.

**Table 10:** Disaster-occurring time corresponding to different Disaster-causing thresholds for underground parking lot A.

Threshold for disaster occurrence		Underground space volume(m^3^)	Disaster-occurring time (h)		
			*P* = 1%	*P* = 2%	*P* = 5%
Common car/child disaster height	0.2	751.6	23.0	24.5	28.3
Disaster height of adult	0.7	2630.6	24.4	25.7	-
Electric box lowest elevation	1.23	4622.3	25.4	27.1	-

Overall, under less intense rainfall (*P* = 5%), the inflow volume into the underground space is considerably lower within the same time period, making it less likely for disaster thresholds to be exceeded. In contrast, under *P* = 1% and *P* = 2% scenarios, the time to reach critical thresholds is concentrated within 25 min, highlighting the urgency of emergency response.

Several “risk intersection points” in the figure—where the capacity curve intersects with disaster threshold lines—indicate the critical inflow volumes and times corresponding to specific flood depths. These points provide a quantitative reference for assessing emergency response time. By integrating spatial geometry, hydraulic processes, and disaster thresholds, the diagram clearly illustrates the underground space’s flood-carrying capacity under different storm intensities. This method offers strong practical value and applicability, particularly for flood risk classification, early warning, and retrofit planning of urban underground infrastructure.

## Conclusion and recommendations

Using advanced surveying equipment specialized for semi-enclosed underground structures (i.e., backpack-mounted 3D laser scanners), precise measurements of complex underground space structures were conducted. This approach enabled the acquisition of entrance/exit attributes and morphological parameters of underground spaces, overcoming the limitations of relying on confidential technical documents such as design drawings and planning maps. Based on the acquired data, a conceptual model of the underground space was constructed to support further analysis.

Based on the surface flood, underground waterlogging, and disaster characteristics of three types of three-dimensional disaster-bearing bodies, an underground space flood indicator system is constructed, and the identification methods for each index are provided. The index system reflects the main information and threshold required for further deduction of the disaster research. As the basis of the disaster characteristics and disaster response of the three-dimensional disaster-bearing body, the establishment of the indicator system of the disaster-bearing body in advance can support the rapid response and further research of external flood and waterlogging. The index system can be adaptively increased or decreased according to the characteristics of the applied city or smaller block unit and the needs of joint prevention and control. The index value can be continuously improved by further supplementing and collecting data, improving measurement, and adopting more reasonable algorithm parameters.

When floodwater invades the underground space, the time required for people to safely evacuate from the underground space consists of five parts. Taking the safe evacuation control water depth at the underground space surface exit as the control condition, the safety evacuation time for a specific underground space can be derived using the typical 3D indicator data obtained. The shortest safety evacuation times for A underground parking lot is 19.5 min. These results can be directly applied to safety assessments. According to the sensitivity analysis results, the exit width has the greatest impact on evacuation time.

The rise rate of water at the surface exit of the underground space significantly affects the safe evacuation of people in the underground space. Based on the constant rise of surface water depth and the water level rise rate in the underground space, the underground flooding depth in A underground parking lots exceeds the surface water depth after approximately 10 min, respectively.

A pedestrian safety-based comprehensive risk assessment method was introduced to develop a Waterlogging Disaster Risk Index (WDR) model that integrates water depth and flow velocity. Damage factors (DF) were classified based on water depth, enabling a multi-dimensional flood risk zoning system. The results show that under the *P* = 5% rainfall scenario, water began to enter the underground space after 27 h, with the WDR value remaining below the high-risk threshold throughout. In contrast, under the *P* = 2% and *P* = 1% scenarios, the WDR exceeded the high-risk level after 28 h and remained elevated for a significantly longer duration. The maximum water depths reached 2.0 m and 3.1 m, respectively, highlighting the increased disaster exposure and evacuation risk of underground spaces under extreme rainfall events. This study provides a physically grounded basis for urban underground flood risk assessment.

The capacity-depth-disaster curve study of the underground space can provide preliminary judgments for decision-making. Future work can involve mutual verification with model simulations, from different perspectives, to deduce the disaster criteria for underground space flooding caused by precipitation.

## Data Availability

All models or datas that support the findings of this study are available from the corresponding author upon reasonable request.
